# Transarterial embolization for chronic wrist pain: a preliminary study of feasibility, safety, and pain reduction

**DOI:** 10.1186/s42155-026-00706-7

**Published:** 2026-05-20

**Authors:** Jeeyoung Min, Jin Ho Hwang, Sang Woo Park, Kwangsun Park, Yun Hak Lee, Woo Young Yang

**Affiliations:** 1 Department of Radiology, Konkuk University Medical Center, 120-1 Neungdong-Ro, Gwangjin-Gu, Seoul, 05030 Republic of Korea; 2 Seoul Sun Orthopedics Clinic, 630 Gonghang-Daero, Yangcheon-Gu, Seoul, Republic of Korea

**Keywords:** Musculoskeletal pain embolization, Transarterial embolization, Chronic wrist pain, Musculoskeletal pain, Quick-soluble gelatin sponge particles

## Abstract

**Background:**

Abnormal neovascularization contributes to chronic musculoskeletal pain. This study aimed to evaluate the preliminary clinical outcomes based on pain reduction of transarterial embolization (TAE) for chronic wrist pain refractory to conservative management.

**Methods:**

This retrospective study analyzed wrist TAE performed for persistent pain caused by triangular fibrocartilage complex (TFCC) injury, de Quervain’s tenosynovitis, or extensor carpi ulnaris (ECU) and flexor carpi ulnaris (FCU) tendinopathy. Selective arterial embolization was performed using temporary gelatin sponge particles based on the anatomical distribution of symptoms. Pain severity was evaluated using the visual analog scale (VAS) at baseline, immediately after treatment, and at 1 day, 1 week, and 1, 3, and 6 months. Technical success was defined as embolization of at least one symptomatic artery. Clinical success was defined as a reduction of more than 50% in VAS score at 6 months.

**Results:**

A total of 12 procedures were performed in 11 patients (median age, 40 years; interquartile range (IQR), 32–45.5). Diagnoses included TFCC injury (*n* = 8), de Quervain’s tenosynovitis (*n* = 2), ECU tendinopathy (*n* = 1), and FCU tendinopathy (*n* = 1). Technical success was achieved in all procedures (100%). The median VAS score decreased from 6.5 (6.0–7.0) at baseline to 1.5 (1.0–3.25) at 6 months (*p* < 0.05). Clinical success was observed in 9 of 12 procedures (75.0%). Regarding safety, all adverse events were low-grade. These included transient erythematous skin reactions (Grade 1) in 58.3% (7/12) of cases, one case of puncture site pseudoaneurysm (Grade 2, 8.3%), and one case of brachial artery dissection (Grade 2, 8.3%). All events resolved without long-term complications.

**Conclusions:**

The results of the current study suggest that TAE is a feasible and well-tolerated treatment option for chronic refractory wrist pain. While these preliminary findings are encouraging, larger prospective studies are warranted to validate clinical effectiveness and define optimal patient selection.

## Background

Transarterial embolization (TAE) has emerged as a minimally invasive treatment for chronic musculoskeletal pain [1–3]. Its efficacy and safety, particularly in the knee joint, have been extensively validated in multiple clinical studies and a recent large-scale meta-analysis of 21 studies [4–8]. Experimental data suggest that TAE suppresses synovial proliferation and inflammatory cell infiltration, providing a therapeutic rationale for its application in other joints that share similar inflammatory and angiogenic pathways [9, 10]. While TAE has shown promise in various anatomical sites, including the shoulder and elbow, clinical evidence specifically for chronic wrist pain remains limited, with existing literature primarily focusing on hand osteoarthritis and finger joints [11–21]. Wrist pain is commonly associated with overuse-related injuries and degenerative conditions, including triangular fibrocartilage complex (TFCC) injury and de Quervain’s tenosynovitis [22, 23]. Although these conditions possess distinct anatomical locations and clinical behaviors, it has been suggested that they may share a common pathophysiological feature: the development of pathological neovascularization during chronic overuse and degenerative change. Based on the shared involvement of neovascularization and associated sensory nerve ingrowth, it was hypothesized that TAE could potentially alleviate symptoms across these varied clinical presentations by targeting a common underlying pain mechanism [9, 10]. Various embolic materials have been reported for TAE, including imipenem–cilastatin, microspheres, and gelatin-based particles [24–27]. Quick-soluble gelatin sponge particles (QS-GSPs), developed to dissolve more rapidly than conventional gelatin sponges, represent a transient arterial occlusion that may reduce the risk of permanent ischemic complications [24, 27, 28].

This exploratory pilot study aimed to assess the feasibility, safety, and preliminary pain outcomes of TAE with QS-GSPs in selected chronic wrist pain conditions sharing a common angiographic target and refractory to conservative management.

## Materials and methods

### Patients

This retrospective study was conducted at a tertiary care center after receiving approval from the institutional review board.

Inclusion and Exclusion Criteria.

The inclusion criteria were as follows:

1. Clinical Chronicity and Severity: chronic wrist pain persisting for at least 6 months, with an initial visual analog scale (VAS) score of ≥ 5.
2. Refractory to Conservative Management: lack of significant clinical improvement despite at least 3 months of conservative treatment. All included patients had undergone a combination of pharmacotherapy (e.g., oral nonsteroidal anti-inflammatory drugs or analgesics), physical therapy (e.g., thermotherapy or electrical stimulation), and local interventions, including corticosteroid injections or extracorporeal shock wave therapy (ESWT). “Refractory” was defined as persistence or recurrence of a VAS score of ≥ 5 after these treatments.
3. Diagnostic Confirmation: a specific diagnosis (e.g., TFCC injury, de Quervain’s tenosynovitis, or ECU/FCU tendinopathy) confirmed by referring orthopedic specialists based on physical examination and ultrasonography.

Preprocedural MRI was not routinely performed because the initial diagnosis at the referring orthopedic clinics was established by clinical assessment and ultrasonography. Angiographic hypervascularity was not used as a formal preprocedural inclusion criterion; rather, it was assessed intraprocedurally to confirm the embolization target.

The exclusion criteria were autoimmune arthropathy, complex regional pain syndrome, systemic inflammatory disease, rheumatoid arthritis, active infection, and prior vascular interventions involving the target wrist. A history of prior wrist arthroscopy was not considered an exclusion criterion, as TAE was also considered in patients with persistent or recurrent symptoms after surgery. During the study period, no patients who met the inclusion criteria were excluded based on these criteria.

### Study population

**Fig. 1:**
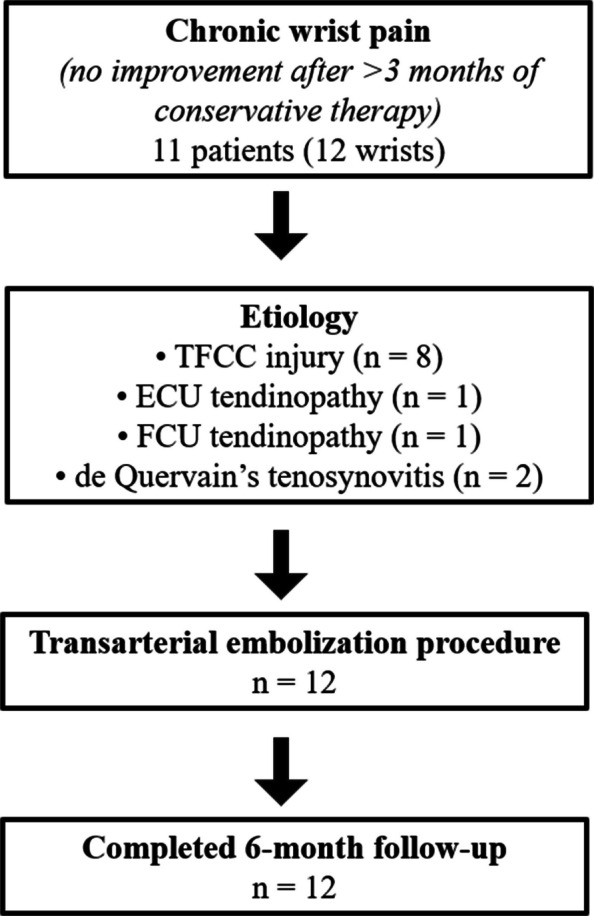
Patient selection and procedure flow diagram. Abbreviations: TFCC, triangular fibrocartilage complex; ECU, extensor carpi ulnaris; FCU, flexor carpi ulnaris

This study reports a consecutive cohort of all patients who underwent wrist TAE and met the inclusion criteria during the initial pilot recruitment period. No patients were excluded on the basis of clinical outcome. Between July 2020 and May 2021, TAE was performed on 12 wrists in 11 patients (Fig. 1). All procedures were performed on an outpatient basis, and patients were discharged on the same day after achieving hemostasis. Detailed baseline characteristics, including age, pain duration, and specific diagnoses, are summarized in the “Results” section and Tables 1 and 2.

**Table 1:** Baseline characteristics of the study population (*n* = 11 patients; 12 procedures)

Characteristic	Value
Sex	Male, 8 (72.7%); female, 3 (27.3%)
Age (years)	Median 40 (IQR, 32–45.5; range, 25–49)
Pain duration (months)	Median 12 (IQR, 12–48; range, 6–120)
Baseline VAS score	Median 6.5 (IQR, 6–7.25; range, 5–9)
Diagnosis	TFCC injury (8); de Quervain’s tenosynovitis (2); ECU tendinopathy (1); FCU tendinopathy (1)
Prior treatments*	Medication (10); physical therapy (1); ESWT (10); steroid or prolotherapy (5)

^*^Patients may have received more than one treatment modality

**Table 2:** Individual patient characteristics and procedural details

Case	Sex/age	Side	Diagnosis	Occupation	Surgical history	Access
1	M/39	L	TFCC injury	Architect	None	TFA
2	F/44	R	ECU tendinopathy	Office worker	None	TFA
3	M/49	R	TFCC injury	Office worker	None	TRA
4	M/25	R	TFCC injury	Photographer	None	TFA
5	M/47	L	de Quervain’s tenosynovitis	Construction worker	None	TFA
6	M/30	R	TFCC injury	Office worker	None	TRA
7	M/25	R	TFCC injury	Student	None	TFA
8	M/25	L	TFCC injury	Student	None	TFA
9	M/34	L	TFCC injury	Baseball player	Arthroscopy	TFA
10	F/47	R	TFCC injury	Homemaker	Arthroscopy	TRA
11	F/41	R	FCU tendinopathy	Office worker	None	TRA
12	M/40	L	de Quervain’s tenosynovitis	Office worker	None	TFA

*Abbreviations*: *TFCC *triangular fibrocartilage complex, *ECU *extensor carpi ulnaris, *FCU *flexor carpi ulnaris, *TFA *transfemoral access, *TRA *transradial access

### Embolization procedure

Arterial access was achieved under local anesthesia via the common femoral artery (CFA; *n* = 7) using a 5-Fr sheath (Radifocus, Terumo, Tokyo, Japan) or the radial artery (TRA; *n* = 4) using a 5-Fr radial sheath (Prelude, Merit Medical, South Jordan, UT, USA). For transradial access (TRA), arterial suitability was confirmed using preprocedural ultrasound and the Barbeau test.

**Fig. 2:**
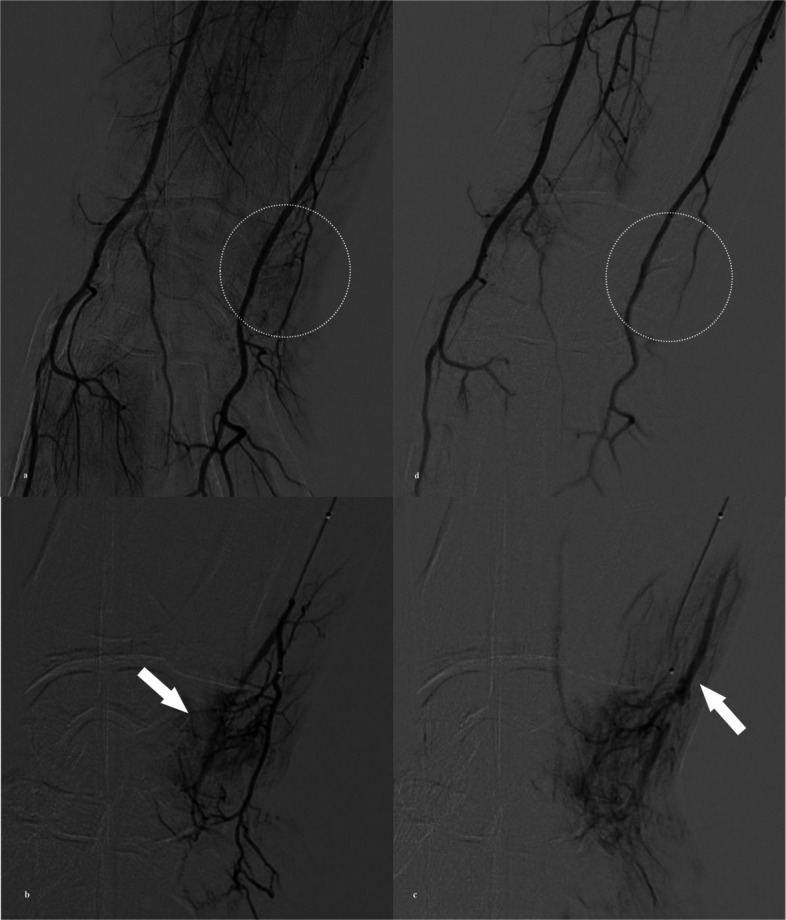
A 44-year-old woman with TFCC injury treated by transcatheter arterial embolization (TAE). **a** Right brachial arteriography via common femoral artery access with a 5-Fr catheter demonstrates hyperstaining on the ulnar side of the wrist joint (white circle). **b** A ulnar artery branch is superselected with a 1.9-Fr microcatheter, showing hypervascular staining corresponding to the pain site (white arrow). **c** Delayed-phase angiography reveals early venous drainage (white arrow) adjacent to the hyperstaining, a finding often observed in TAE and considered an additional marker for embolization. **d** Final angiography after injection of 0.5 mL quick-soluble gelatin sponge particles (QS-GSPs) demonstrates resolution of the hyperstaining (white circle). The VAS pain score improved from 7 at baseline to 1 at 6 months

**Fig. 3:**
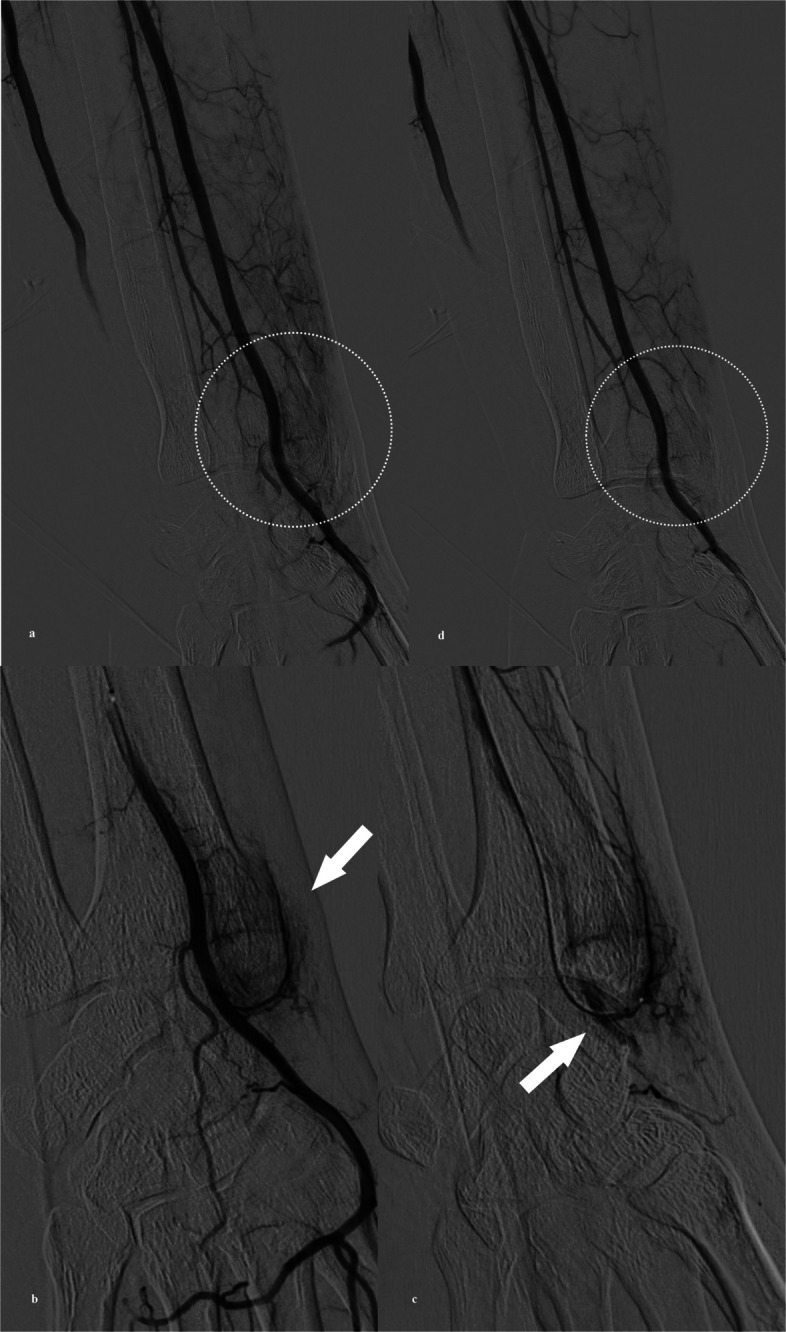
A 40-year-old man with de Quervain’s tenosynovitis. **a** Left brachial arteriography via common femoral artery access with a 4-Fr catheter shows hyperstaining on the radial side of the wrist joint (white circle). **b** A 1.9-Fr microcatheter demonstrates hypervascular staining at the pain site (white arrow). **c** Delayed-phase angiography after superselection of a radial artery branch reveals early venous drainage (white arrow) adjacent to the hyperstaining. **d** Final angiography after injection of 1.6 mL QS-GSPs shows resolution of the hyperstaining (white circle). The VAS pain score improved from 8 at baseline to 1 at 6 months

A 4-Fr Glidecath (Terumo) for CFA access or a 5-Fr Omni-flush catheter (AngioDynamics, Queensbury, NY, USA) for TRA was positioned in the brachial artery via either approach, and baseline angiography was performed. This was used to identify focal hypervascular staining corresponding to the patient’s symptomatic wrist area (Figs. 2 and 3). In all cases, the angiographic blush spatially matched the clinical site of tenderness and was used to guide target vessel selection.

Once the target was identified, superselective catheterization of the feeding vessel was conducted using a 1.9-Fr microcatheter (Tellus; Asahi Intecc, Nagoya, Japan) and a 0.016-inch microguidewire (Meister, Asahi Intecc) (Figs. 2 and 3). QS-GSPs (IPZA, Engain, Gyeonggi-do, Republic of Korea; 50–150 µm, mean 90 µm) were used as a temporary embolic agent in all procedures. These rapidly biodegradable gelatin-based particles achieve approximately 90% volume dissolution within 24 h in vitro, thereby minimizing the risk of permanent ischemic complications in small periarticular vessels. The particles were prepared with a 2:8 mixture of saline and iodinated contrast (Visipaque, GE Healthcare) and injected until near flow stasis and resolution of hyperstaining were achieved.

Final angiography was performed to confirm the absence of residual hypervascular staining (Figs. 2 and 3). Hemostasis was achieved using a vascular closure device (Mynx; Cordis, Miami Lakes, FL, USA) for CFA access or a radial compression device (PreludeSYNC, Merit Medical Systems) for TRA. Patients were discharged on the same day after a short observation period.

### Assessment and follow-up

Clinical and imaging data were retrieved from electronic records, and image archiving and communication systems were reviewed. Follow-up assessments were conducted either during scheduled outpatient visits or telephone contact. Outpatient follow-up was primarily performed at the referring orthopedic clinic, where one orthopedic surgeon and two interventional radiologists participated in patient evaluation. Changes in wrist pain after the procedure and the occurrence of adverse events (AEs) were recorded (Table 3). Pain intensity was assessed using VAS, reflecting overall wrist pain including both pain at rest and pain during wrist movement (0 = no pain, 10 = maximum pain intensity). VAS scores were assessed at baseline, immediately after procedure, and at one day, one week, one month, three months, and six months after the procedure. Clinical success was defined as a > 50% reduction in the VAS score from baseline at 6-month follow-up. Adverse events were classified according to the modified Cardiovascular and Interventional Radiological Society of Europe (CIRSE) classification system for complications [29, 30]. Events such as skin color change at the treatment site, puncture-site hematoma, muscle weakness, and paralysis were classified by severity and clinical outcome using this system.

**Table 3:** Clinical outcomes and adverse events

Case	Baseline VAS	6-month VAS	Clinical success	Adverse events	CIRSE grade
1	6	1	Yes	None	–
2	7	1	Yes	Skin erythema	1
3	6	1	Yes	Skin erythema	1
4	9	4	Yes	Skin erythema	1
5	6	1	Yes	None	–
6	7	1	Yes	Skin erythema	1
7	5	1	Yes	CFA pseudoaneurysm	2
8	7	2	Yes	Skin erythema	1
9	6	4	No	None	–
10	5	3	No	Skin erythema	1
11	7	4	No	Brachial artery dissection	2
12	8	1	Yes	Skin erythema	1

Clinical success was defined as a reduction of ≥ 50% in VAS score at 6 months compared with baseline

– indicates no adverse event

*Abbreviations*: *VAS *visual analog scale, *CIRSE *Cardiovascular and Interventional Radiological Society of Europe

### Statistical analysis

The data were not normally distributed at several time points (as assessed by the Shapiro–Wilk test), and a non-parametric Friedman test was therefore conducted to evaluate the overall differences in VAS scores over time. Pairwise comparisons between time points were conducted using the Wilcoxon signed-rank test with the Bonferroni correction for multiple comparisons. All statistical analyses were performed using Python 3.11 with the scipy and pandas packages (Python Software Foundation, Wilmington, DE, USA). Graphs were created using Matplotlib (https://matplotlib.org) and Seaborn (https://seaborn.pydata.org) software.

## Results

### Patient characteristics

A total of 12 procedures were performed in 11 patients (eight men and three women; seven right and five left wrist joints). The median age was 40 years (interquartile range (IQR), 32–45.5; range, 25–49 years). The median pain duration prior to the procedure was 12 months (IQR, 12–48 months; range, 6–120 months), with a mean duration of 33.0 ± 33.9 months. All patients met the inclusion criteria of having chronic refractory pain with a baseline VAS score ≥ 5. The baseline demographics, clinical characteristics and procedural details are provided in Tables 1 and 2.

### Angiographic findings and technical success

Technical success was achieved in all 12 procedures (100%). In each case, baseline angiography confirmed the presence of pathological hypervascular staining that correlated precisely with the clinical site of tenderness. In the 10 procedures performed for ulnar-sided wrist pain (TFCC injury, *n* = 8; FCU/ECU tendinopathy, *n* = 2), the feeding vessels originated from the ulnar artery branches at the wrist level (Fig. 2). In the two cases of radial-sided pain due to de Quervain’s tenosynovitis (*n* = 2), the feeders originated from the radial artery branches (Fig. 3). The mean volume of QS-GSPs injected per procedure was 1.2 ± 0.6 mL.

### Clinical outcomes

**Fig. 4:**
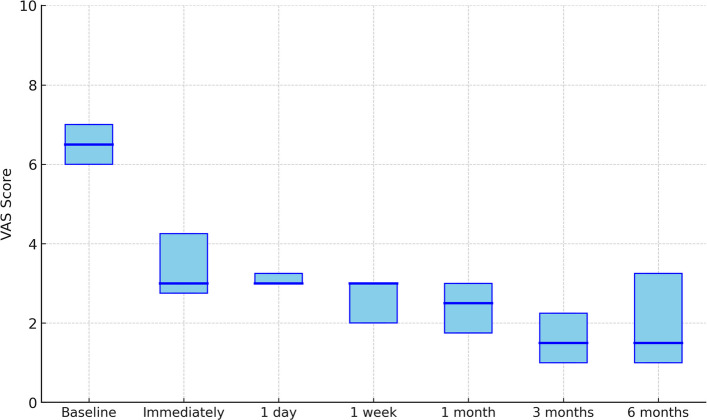
Boxplots showing the distribution of VAS pain scores at each time point (baseline, immediately, 1 day, 1 week, 1 month, 3 months, and 6 months). Median values are indicated by horizontal lines, with boxes representing the interquartile range. The median VAS scores at baseline, immediately after TAE, and at 1 day, 1 week, 1 month, 3 months, and 6 months were 6.5 (6.0–7.0), 3.0 (2.75–4.25), 3.0 (3.0–3.25), 3.0 (2.0–3.0), 2.5 (1.75–3.0), 1.5 (1.0–2.25), and 1.5 (1.0–3.25), respectively (all *p* < 0.05)

All 11 patients completed the 6-month follow-up, with a median follow-up duration of 6.1 months (5.7–6.9). The median VAS scores at baseline, immediately after TAE, and at 1 day, 1 week, 1 month, 3 months, and 6 months were 6.5 (6.0–7.0), 3.0 (2.75–4.25), 3.0 (3.0–3.25), 3.0 (2.0–3.0), 2.5 (1.75–3.0), 1.5 (1.0–2.25), and 1.5 (1.0–3.25), respectively (all *p* < 0.05) (Fig. 4). These showed a statistically significant decrease over time (Friedman test, *χ*^2^(6) = 51.01, *p* < 0.001). Post hoc Wilcoxon signed-rank tests with Bonferroni correction demonstrated that all post-treatment time points—immediately, 1 day, 1 week, 1 month, 3 months, and 6 months—had significantly lower VAS scores compared to baseline (*p* < 0.05). Specifically, the median VAS decreased from 6.5 (6.0–7.0) at baseline to 1.5 (1.0–3.25) at 6 months.

The overall clinical success rate at 6 months was 75% (9/12 procedures). Due to the pilot nature of this study and the heterogeneous diagnostic entities, subgroup results were analyzed descriptively. Clinical success was observed in 75% (6/8) of TFCC injuries, 100% (2/2) of de Quervain’s tenosynovitis, and 100% (1/1) of ECU/FCU tendinopathy cases (Table 3). A comparative description between the clinical success group (*n* = 9) and the failure group (*n* = 3) revealed distinct characteristics. Of the three clinical failures, two (66.7%) had a history of prior wrist surgery (arthroscopy), whereas only one patient (11.1%) in the success group had a surgical history. Furthermore, the failure cases were characterized by complex TFCC injuries with high baseline VAS scores (≥ 7) and a more chronic clinical course.

### Safety outcomes

**Fig. 5:**
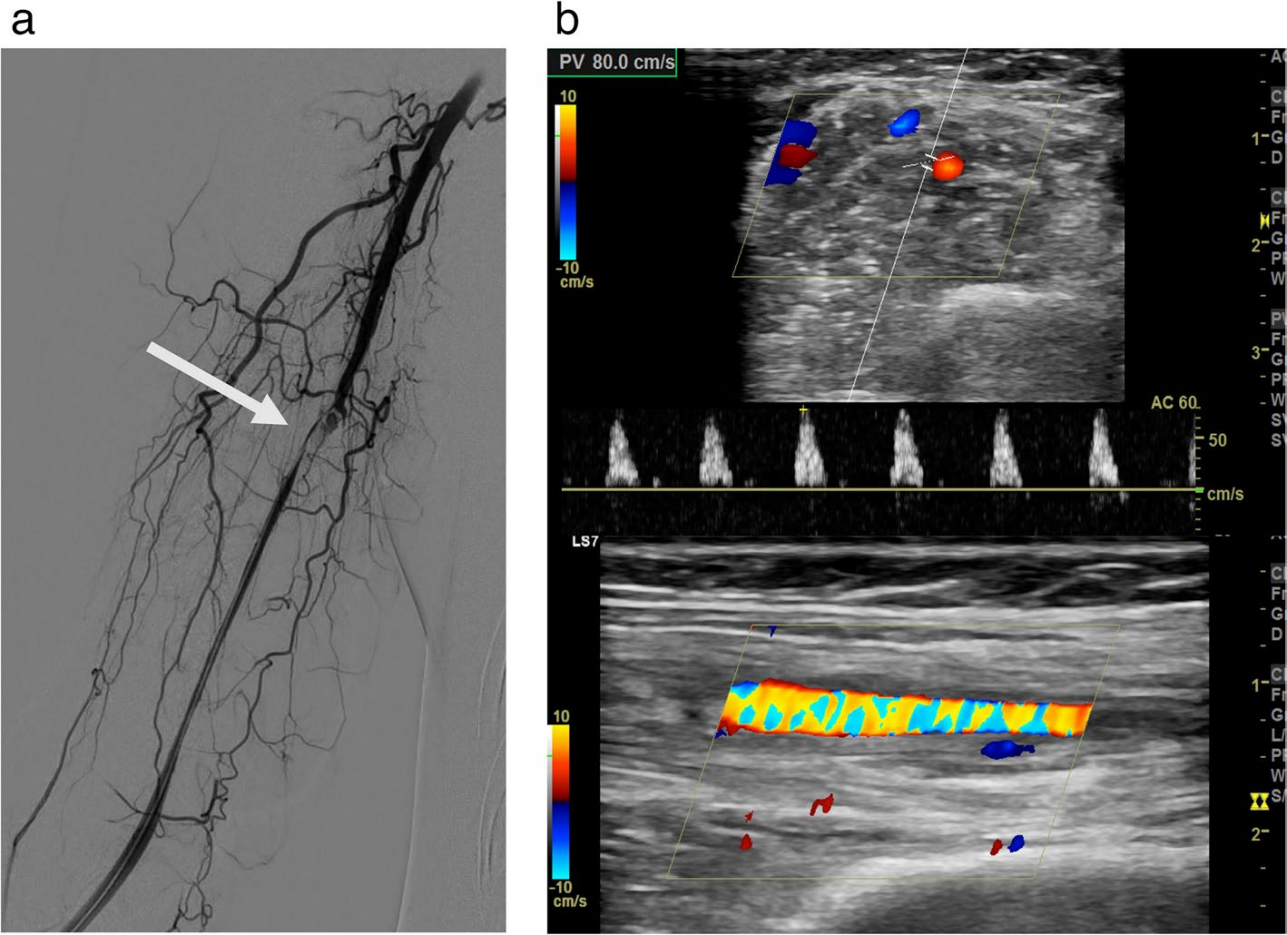
**a** A 41-year-old woman underwent TAE for right wrist pain. The right radial artery was punctured, and a 5-Fr OmniFlush catheter was advanced retrogradely. Embolization of the target feeder was completed, although contrast stasis was observed. Final angiography demonstrated a dissection in the mid-brachial artery (white arrow), likely related to catheter manipulation, with distal flow preserved. **b** Postprocedural ultrasound revealed mild external compression by a hematoma, with Doppler flow maintained. Follow-up ultrasound at 1 week confirmed complete resolution, and the patient remained asymptomatic

All AEs were classified according to the CIRSE classification system [29, 30]. Transient erythematous skin reactions (Grade 1) resembling livedo reticularis were observed in seven patients (58.3%), and resolved spontaneously without treatment within one week. One case of pseudoaneurysm (Grade 2) at the puncture site of the common femoral artery was confirmed using ultrasonography and resolved after compression. One case of brachial artery dissection (Grade 2) occurring during catheter manipulation resulted in a small hematoma without flow restriction, which was managed conservatively, and complete recovery was confirmed after one week (Fig. 5). No major complications (Grade 3 or higher), such as muscle weakness, paralysis, or anaphylaxis were observed (Table 3).

## Discussion

In the present study, TAE was extended to chronic wrist pain refractory to conservative treatment. The underlying mechanism is presumed to parallel that of knee TAE, in which embolization of hypervascular synovitis suppresses inflammatory cell infiltration and aberrant sensory nerve ingrowth [8–10]. In our cohort, the areas of hypervascular staining on angiography consistently corresponded to the clinical sites of pain and the pathological findings identified on preprocedural ultrasonography. These findings suggest that pathological neovascularization contributes to chronic pain across various wrist conditions, including both degenerative and overuse-related injuries such as TFCC tears and de Quervain’s tenosynovitis.

Our clinical success rate of 75% is consistent with reported outcomes of TAE in other upper extremity musculoskeletal conditions. Previous studies on adhesive capsulitis of the shoulder and both lateral and medial epicondylitis of the elbow have demonstrated clinical success rates ranging from 73 to 86% using various embolic agents. These similarities across different upper-extremity sites support the hypothesis that pathological neovascularization may represent a shared therapeutic target in selected chronic musculoskeletal pain conditions. However, despite this shared angiographic target, underlying pathophysiological differences among these conditions may result in variable treatment responses.

In this exploratory study, TAE with QS-GSPs demonstrated favorable pain outcomes in patients with chronic wrist pain. Although individual responses varied across the heterogeneous diagnostic entities, subgroup results showed clinical success in 75% of TFCC injuries and 100% of other tendinopathies. Three patients did not experience meaningful pain reduction; notably, two of these had a history of prior wrist surgery. It is plausible that postsurgical anatomical alterations or chronic scarring limited the efficacy of TAE or affected the angiographic identification of pathological hypervascularity. These findings suggest that prior surgical intervention and advanced structural chronicity may be associated with a limited therapeutic response to TAE. This observation underscores the importance of appropriate patient selection, particularly in patients with prior surgical history and advanced structural changes.

Of the 12 procedures, radial artery access was conducted in four cases. TRA offers several advantages over transfemoral access, including reduced vascular complications, earlier ambulation, and greater patient comfort [31]. In this study, TRA was selected only in cases where the target feeder originated from the ulnar artery, the patient preferred earlier ambulation and discharge, and preprocedural ultrasonography confirmed a radial artery diameter ≥ 2 mm with normal Doppler waveforms. Prior studies have validated QS-GSPs as effective transient embolic materials for musculoskeletal conditions [24–27]. In this study, they were selected to achieve transient occlusion of pathological neovascularity while minimizing ischemic risk to the distal tissues. Although this specific agent is currently available in a limited regional market, its mechanism is comparable to other resorbable embolic materials used internationally [24]. Previous studies have demonstrated consistent clinical outcomes across different temporary embolic platforms, supporting the generalizability of our findings [24].

Despite their rapid dissolution, the observed durability of pain relief suggests that even transient ischemia may induce regression of (pain-related) unmyelinated sensory nerve fibers, thereby interrupting the cycle of neurogenic inflammation [9, 10].

The particle size used in this study (50–150 µm; mean 90 µm) may have contributed transient cutaneous reactions; however, all events were self-limited and resolved without sequelae, supporting the safety profile of temporary embolic agents.

This study has several limitations. Firstly, the retrospective nature and small sample size, combined with the diagnostic heterogeneity of the cohort, may limit the internal validity and generalizability of the outcomes to each specific condition. The small cohort size primarily reflects the challenges inherent in recruiting for an early-phase pilot study of an unfamiliar intervention. Despite this, the cohort includes all consecutive consenting patients within the defined period without any outcome-based exclusion, thereby minimizing the risk of selective inclusion.

Regarding diagnostic precision, the diagnosis of TFCC injury in this cohort was based on clinical examination by an orthopedic specialist and ultrasonography, without routine MRI or arthrographic confirmation, as these modalities were not available at the referring institution. While this reflects a common diagnostic pathway in community-based orthopedic practice, ultrasonography alone may not fully characterize the structural extent of TFCC injury, and this represents a meaningful diagnostic limitation that should be considered when interpreting outcomes in this subgroup.

Secondly, clinical outcomes were primarily assessed using VAS scores without standardized functional measures, such as the Quick Disabilities of the Arm, Shoulder, and Hand (QuickDASH) or Patient-Rated Wrist Evaluation (PRWE) scores.

Furthermore, the absence of pre- and post-procedural MRI in all patients limits the objective evaluation of subclinical structural changes or subtle functional impairments. As an exploratory pilot study, these results should be interpreted as a preliminary signal of technical feasibility and potential efficacy. Future prospective, disease-specific trials with larger cohorts, standardized imaging, and validated functional outcome tools are required to establish the precise role of TAE for each diagnostic category.

## Conclusion

In this exploratory pilot study, TAE with temporary gelatin sponge particles was technically feasible and safe, with favorable preliminary pain reduction in selected patients with chronic refractory wrist pain. Given the small and diagnostically heterogeneous cohort, as well as the absence of standardized functional outcome measures, these findings should be interpreted with caution. Further prospective studies are warranted to establish clinical efficacy and optimize patient selection.

## Data Availability

The datasets generated and analyzed during the current study are available from the corresponding author on reasonable request.

## Abbreviations

TAE: Transarterial embolization
VAS: Visual analog scale
TFCC: Triangular fibrocartilage complex
ECU: Extensor carpi ulnaris
FCU: Flexor carpi ulnaris
QS-GSPs: Quick-soluble gelatin sponge particles
ESWT: Extracorporeal shock wave therapy
CFA: Common femoral artery
TRA: Transradial access
AEs: Adverse events
CIRSE: Cardiovascular and Interventional Radiological Society of Europe
QuickDASH: Quick Disabilities of the Arm, Shoulder, and Hand
PRWE: Patient-Rated Wrist Evaluation
